# Differentiated ovine tracheal epithelial cells support the colonisation of pathogenic and non-pathogenic strains of *Mannheimia haemolytica*

**DOI:** 10.1038/s41598-020-71604-8

**Published:** 2020-09-11

**Authors:** Nicky O’Boyle, Catherine C. Berry, Robert L. Davies

**Affiliations:** 1 Institute of Infection, Immunity and Inflammation, College of Medical, Veterinary and Life Sciences, University of Glasgow, Glasgow, UK; 2 Institute of Molecular, Cell and Systems Biology, College of Medical, Veterinary and Life Sciences, University of Glasgow, Glasgow, UK

**Keywords:** Bacterial pathogenesis, Bacterial physiology, Cellular microbiology, Cellular imaging, Mechanisms of disease, Microbiology, Bacteria, Bacteriology, Biofilms, Cellular microbiology, Pathogens

## Abstract

*Mannheimia haemolytica* is the primary bacterial species associated with respiratory disease of ruminants. A lack of cost-effective, reproducible models for the study of *M. haemolytica* pathogenesis has hampered efforts to better understand the molecular interactions governing disease progression. We employed a highly optimised ovine tracheal epithelial cell model to assess the colonisation of various pathogenic and non-pathogenic *M. haemolytica* isolates of bovine and ovine origin. Comparison of single representative pathogenic and non-pathogenic ovine isolates over ten time-points by enumeration of tissue-associated bacteria, histology, immunofluorescence microscopy and scanning electron microscopy revealed temporal differences in adhesion, proliferation, bacterial cell physiology and host cell responses. Comparison of eight isolates of bovine and ovine origin at three key time-points (2 h, 48 h and 72 h), revealed that colonisation was not strictly pathogen or serotype specific, with isolates of serotype A1, A2, A6 and A12 being capable of colonising the cell layer regardless of host species or disease status of the host. A trend towards increased proliferative capacity by pathogenic ovine isolates was observed. These results indicate that the host-specific nature of *M. haemolytica* infection may result at least partially from the colonisation-related processes of adhesion, invasion and proliferation at the epithelial interface.

## Introduction

Respiratory disease of ruminants is a multifactorial process that represents a fascinating example of host–pathogen interactions. Stress and immune status of the animal contribute to susceptibility to infection with opportunistic viral and bacterial pathogens, leading to significant levels of morbidity and mortality^1^. *Mannheimia haemolytica* is a common resident of the upper respiratory tract of healthy cattle and sheep. At the onset of respiratory disease, viral infection or suppression of the immune system results in proliferation of *M. haemolytica* in the bronchioles and alveoli leading to extensive tissue necrosis in an outcome termed pneumonic pasteurellosis^1^. Less prevalent bacterial causes of respiratory disease in livestock include *Pasteurella multocida* and *Histophilus somni*, while viral agents of respiratory disease complex include bovine respiratory syncytial virus, bovine herpesvirus, bovine diarrheal virus and bovine coronavirus^2–4^. Bovine respiratory disease represents a major economic burden to the agriculture industry with losses of up to $1 billion annually in the United States of America^5^. While the majority of studies on respiratory disease of ruminants have focused on cattle, sheep and other ruminant livestock suffer similar infections, also having significant economic impact. For example, losses due to ovine pneumonia in New Zealand in 2008 were $28.1 million^6^.

The virulence factor repertoire of *M. haemolytica* has yet to be well-defined. A lack of readily available molecular tools for genetic manipulation of the organism coupled with a lack of economically viable infection models has hampered efforts to characterise pathogenesis at a molecular level. Observations from infected animals have, however, been somewhat enlightening. In healthy cattle for example, non-pathogenic isolates of predominantly serotype A2 occupy the nasopharynx while cattle suffering from respiratory disease on the other hand, become heavily colonised by serotype A1 or A6 isolates^7–10^. These observations have led to the conclusion that serotype A1 and A6 strains are pathogenic in cattle whereas serotype A2 strains are considered non-pathogenic and a commensal of the normal respiratory flora^9,11,12^. By contrast sheep displaying respiratory disease become heavily colonised by *M. haemolytica* serotype A2, while diverse serotypes such as A5, A9, A12 and A13 are more rarely associated with disease^13–15^. However, it is important to appreciate that genetically distinct sub-populations of *M. haemolytica* isolates of serotypes A1, A2 and A6 are associated with disease and carriage in cattle and sheep and that capsular serotype is not, by itself, an indicator of host-specificity and virulence^16^. Nevertheless, these observations indicate interesting host-specific and strain-specific virulence traits in *M. haemolytica*. Serotyping of *M. haemolytica* is dependent upon variation in the polysaccharide capsule that is thought to play a role in adhesion to host cells^17^ and evasion of phagocytosis by macrophages and neutrophils^18^. Surface proteins such as OmpA and Lpp1 have also been shown to play a role in initial colonisation^19^. Transferrin binding proteins TbpA and TbpB^20^ and a 340 kDa filamentous haemagglutinin^11^ have also been proposed to play a role in this process based on surface expression and homology with adhesins in other species, although their roles in *M. haemolytica* adhesion have not been experimentally confirmed. In addition to colonisation and proliferation, excessive stimulation of the immune system is a hallmark of *M. haemolytica* pathogenesis. Lipopolysaccharide (LPS) causes the activation of proinflammatory cytokine secretion from leukocytes^21^, while secretion of leukotoxin (encoded by *lktA*)—widely regarded as the primary virulence factor of *M. haemolytica*—leads to degranulation and apoptosis of neutrophils, greatly contributing to the inflammatory necrosis associated with ruminant respiratory disease^22,23^. In recent years, there has been a growing interest in the development of cost-effective, biologically relevant models to gain a more complete understanding of the molecular mechanisms underlying these pathogenic processes and the factors determining host-specificity of *M. haemolytica*.

With this in mind, our group have developed highly optimised in vitro tissue engineered models of both cattle and sheep respiratory epithelia^24–27^. By optimising medium, growth factor concentrations, substrate pore density, atmospheric gas composition and period of differentiation, these models best reflect the thickness, ciliation level, mucus production and cellular composition of their source of isolation. These models provide an excellent platform for the study of initial interactions between *M. haemolytica* and the respiratory epithelium. We have shown that differentiated bovine bronchial epithelial cells (BBECs) support the colonisation of pathogenic *M. haemolytica*^28^. In this study, we aimed to assess whether differentiated ovine tracheal epithelial cells (OTECs) displayed similar pathogen-specific colonisation. Through the assessment of a broader collection of pathogenic and non-pathogenic isolates of known genetic relatedness^16^ and host origin, we also assessed whether the ovine model displayed selectivity towards bovine and ovine isolates. Surprisingly, we did not observe a strict pathotype or host-origin selectivity using this model, but the physiology of both tissue-adherent bacteria and the colonised host epithelia were different comparing pathogenic and non-pathogenic *M. haemolytica* isolates. These findings have implications for future use of the model for the study of commensal respiratory species and agents of the respiratory disease complex.

## Results

**Figure 1:**
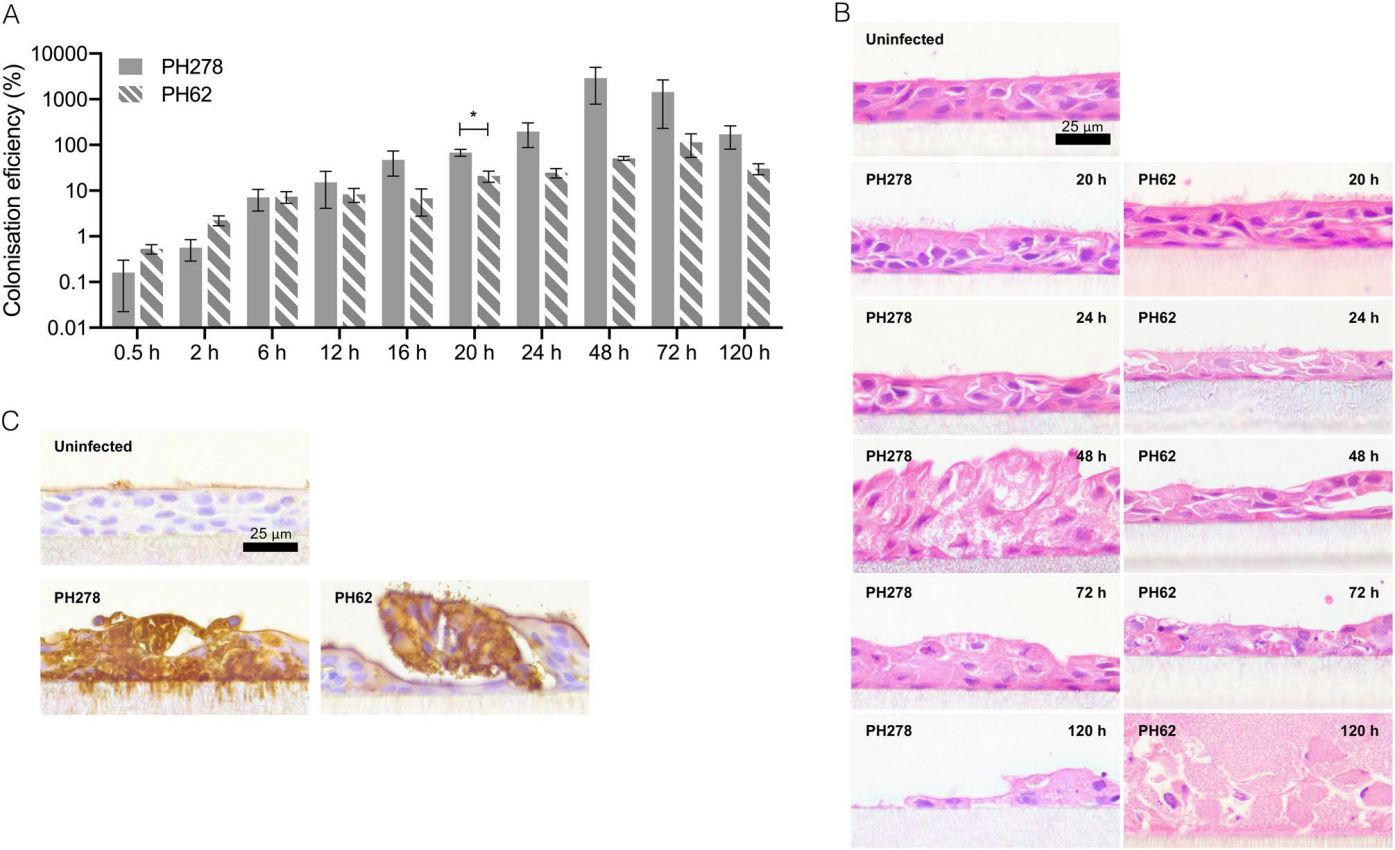
Pathogenic and non-pathogenic isolates of *M. haemolytica* display differential dynamics of colonisation on differentiated OTECs. Differentiated OTECs were infected with either pathogenic (PH278) or non-pathogenic (PH62) *M. haemolytica* isolates for the indicated periods of time. Non-adherent bacteria were removed by washing and tissues were processed as follows. (**A**) Tissues were lysed by addition of 1% (v/v) Triton X-100 and bacteria were enumerated by serial dilution and spot plating. The numbers of bacteria in the lysate were expressed as a percentage of the inoculum (colonisation efficiency [%]). Columns display means of three experiments +/− standard error, with triplicate inserts being used per experiment. Statistical significance was assessed at each independent time-point by two-tailed unpaired Student’s *t* test, with * representing *p* < 0.05. (**B**) Histological sections demonstrating proliferation of PH278 and PH62 within differentiated OTECs. Tissues were fixed in 4% (w/v) paraformaldehyde, infiltrated with paraffin resin, sectioned and stained with haematoxylin and eosin (H&E) as outlined in methods. (**C**) Immunohistochemical labelling of PH278- and PH62-infected OTECs. At 24 h, tissues were fixed in 4% (w/v) paraformaldehyde, infiltrated with paraffin resin, sectioned and immunostained with anti-OmpA^PH278^ (brown) and counterstained with Gill’s haematoxylin.

In order to allow the study of *M. haemolytica* disease progression in vitro, it was important that our model support the colonisation of disease-causing strains. We first assessed the colonisation of differentiated OTECs by PH278 and PH62, which represent the major ovine disease-associated serotype (A2) and a serotype rarely associated with disease in sheep (A12), respectively (Table 1). Strain PH278 was isolated from the lungs of a sheep with pneumonic pasteurellosis, while PH62 was isolated from the nasopharynx of a healthy sheep. Both isolates were capable of colonising the differentiated OTEC layer with varying dynamics of growth on the tissue surface. In general, there was a trend of increased initial adhesion and early colonisation (0.5, 2 and 6 h) by PH62, with higher mean levels of adhesion being observed for PH278 at all time-points after 6 h. However, it should be noted that a statistically significant difference between the strains was only observed at 20 h (Fig. 1A). Colonisation levels equating to 2,891% of the inoculum were observed for PH278 at 48 h indicative of extensive proliferation. This was followed by a reduction to 171% by 120 h as the excessive bacterial proliferation led to sloughing of the cell layer during washing. By contrast, at 48 h, colonisation by PH62 reached only 51% of the inoculum indicating comparatively poor proliferation. There was a reasonably high level of consistency between experiments with overlap being observed in colonisation efficiency between tissues isolated from independent animals at the majority of time-points (Fig. S1), indicating that the model provides a high level of reproducibility.

**Table 1:** Properties of bacterial strains.

Strain	Reference isolates^a^	Host species	Disease status	Site of origin	ET^b^	Capsular serotype	LPS-type^c^	OMP-type^d^	*lktA *allele^e^	*ompA *allele^f^	*tbpBA *allele^g^
PH2	PH101	Bovine	Pneumonia	Lung	1	A1	1A	1.1.1	*lktA1.1*	*ompA1.1*	*tbpBA1.1*
PH376	PH105	Bovine	Pneumonia	Lung	1	A6	1A	1.1.4	*lktA1.1*	*ompA1.1*	*tbpBA1.1*
PH62^h^	PH110	Ovine	Healthy	Nasopharynx	1	A12	1A	1.2.3	ND	ND	ND
PH346	PH110	Ovine	Unknown	Unknown	1	A12	1A	1.2.3	*lktA1.2*	*ompA2.1*	*tbpBA2.1*
PH202	PH2101	Bovine	Healthy	Nasopharynx	21	A2	3B	2.1.2	*lktA2.2*	*ompA1.3*	*tbpBA7.2*
PH210	PH2102	Bovine	Healthy	Nasopharynx	21	A2	3B	2.1.3	*lktA2*	ND	ND
PH278	PH2104	Ovine	Pneumonia	Lung	21	A2	3B	2.2.2	*lktA10.1*	*ompA2.3*	*tbpBA1.8*
PH372	PH2104	Ovine	Septicaemia	Lung	21	A2	3B	2.2.2	*lktA10.1*	*ompA2.3*	*tbpBA1.8*

^a^Reference isolates identify ET strain groups, and associated characteristics, to which isolates belong as defined in reference Davies et al*.*^16^.

^b^ET, electrophoretic type as defined in reference Davies et al*.*^16^.

^c^LPS-type, lipopolysaccharide-type as defined in reference Davies and Donachie^44^.

^d^OMP-type, outer membrane protein-type as defined in reference Davies and Donachie^44^.

^e^*lktA* alleles, as defined in reference Davies et al*.*^39^.

^f^*ompA* alleles, as defined in reference Davies et al*.*^39^.

^g^*tbpBA* alleles as defined in reference Lee and Davies^31^.

^h^Isolate PH62 = NCTC 10644, all other strains were field isolates obtained from University of Glasgow strain collection or Veterinary Investigation Centres (UK).

Extensive tissue disruption was observed by haematoxylin and eosin (H&E) staining from 48 h with PH278 and from 72 h with PH62 (Fig. 1B). Regions of nuclear condensation and cell-sloughing were evident at these time-points. While bacteria were difficult to visualise in H&E-stained sections, they could be readily observed at 24 h by immunohistochemistry using an anti-OmpA^PH278^ antibody (Fig. 1C). OmpA is a good candidate for detection of multiple strains due to its high conservation in *M. haemolytica*^29^. Strikingly, at 120 h post-infection in some sections PH62 displayed large regions of bacterial growth greater than 50 μm deep and 200 μm wide (Fig. 1B), consistent with microcolony formation. PH278 on the other hand did not form microcolonies and instead appeared to completely disrupt the tissue layer by this time-point, leading to flooding of the apical chamber and growth of bacteria on the cell culture insert, while PH62-infected cell layers remained intact.

**Figure 2:**
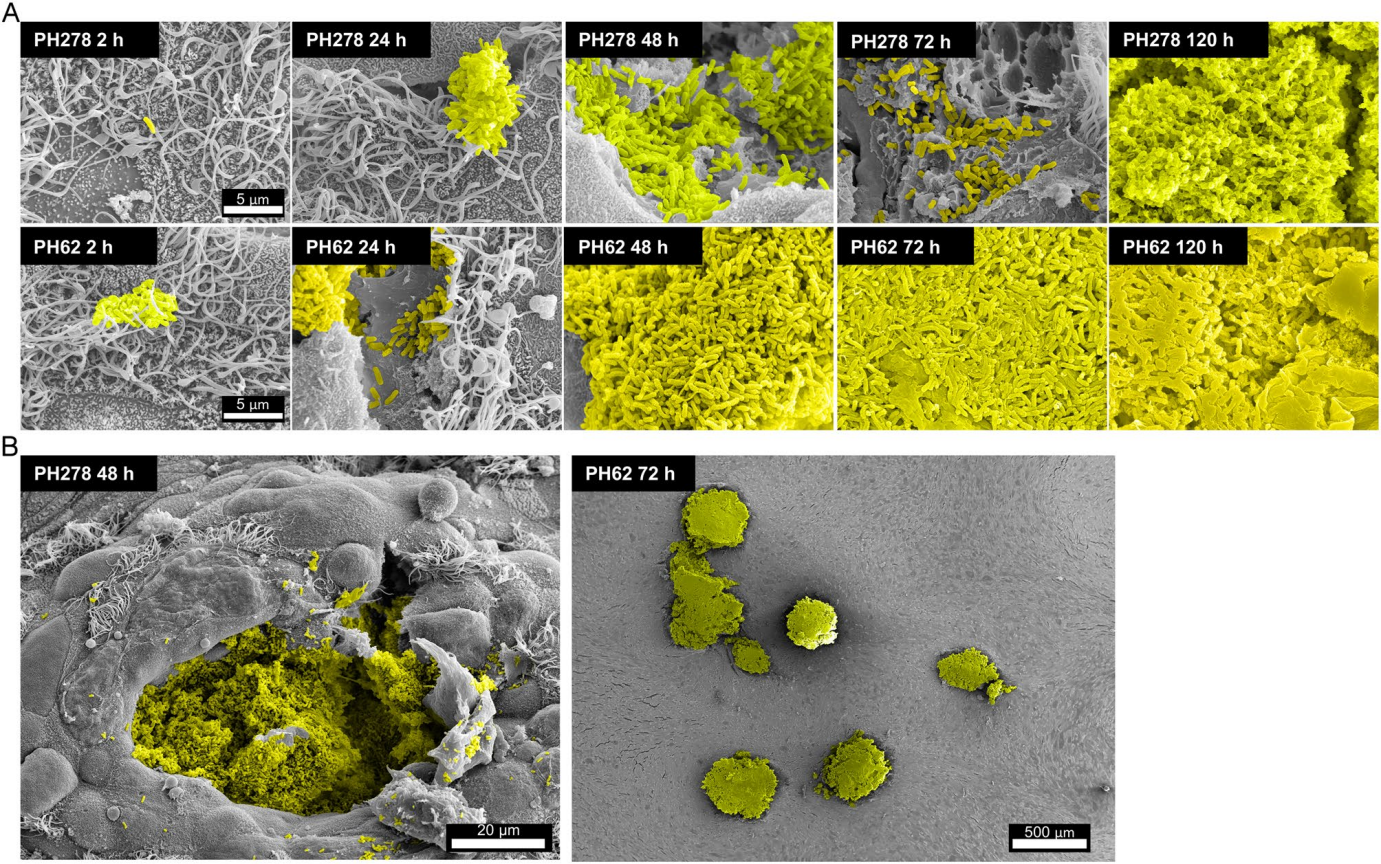
Scanning electron microscopy (SEM) reveals distinct patterns of colonisation of differentiated OTECs for PH278 and PH62. Differentiated OTECs were infected with either pathogenic (PH278, serotype A2) or non-pathogenic (PH62, serotype A12) *M. haemolytica* isolates for the indicated periods of time. Non-adherent bacteria were removed by washing and tissues were fixed and processed for SEM. Bacteria were false-coloured yellow to aid in visualisation. (A) High magnification images showing the interactions of single *M. haemolytica* cells with host tissues at various time-points. All images in panel A were acquired at identical magnification. (B) Low magnification images showing distinguishing characteristics of PH278 and PH62 infection. PH278 appears to invade the epithelial cell layer, causing the rounding and sloughing of surrounding cells, while PH62 forms discrete microcolonies approximately 300 μm in diameter that are encased in amorphous material.

**Figure 3:**
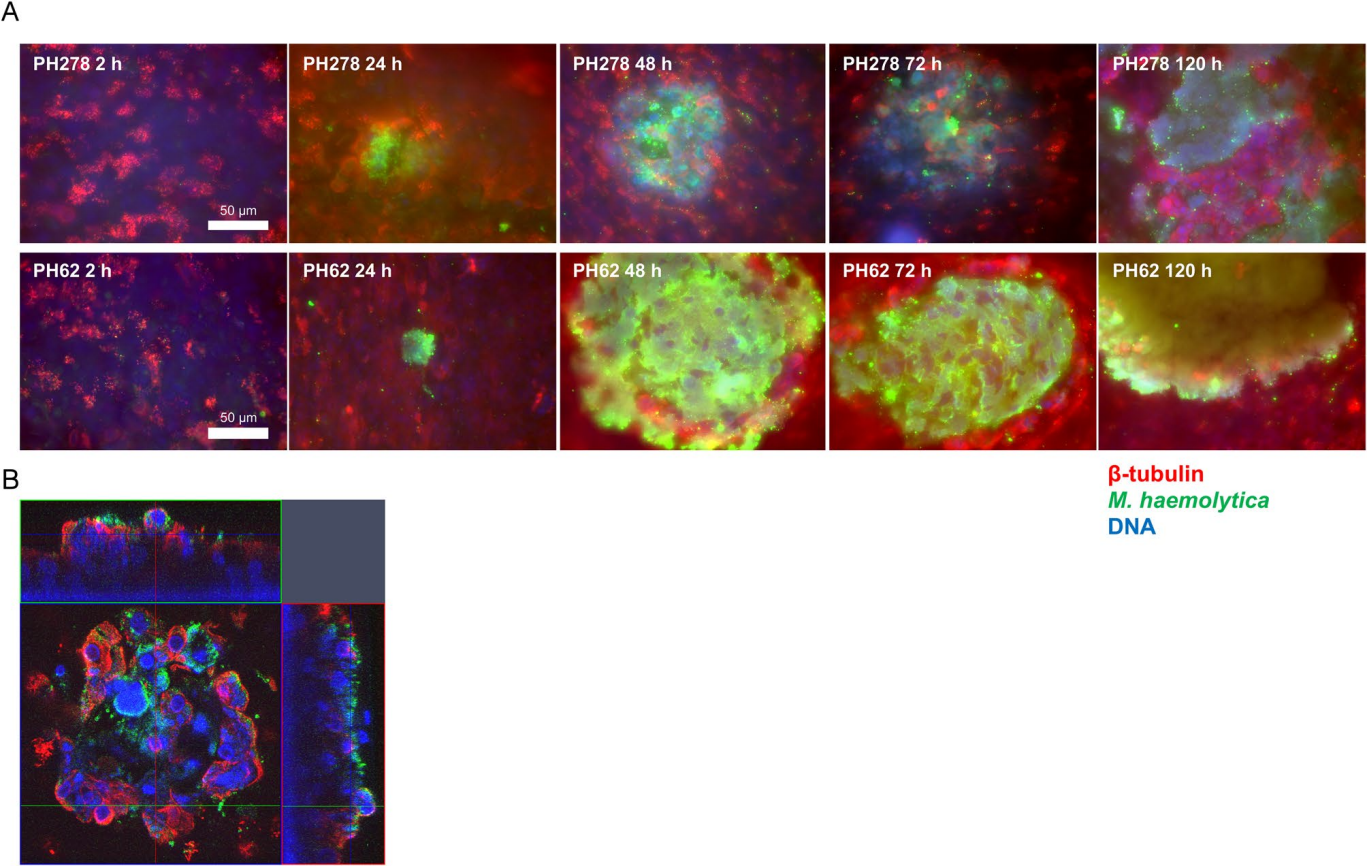
Immunofluorescence microscopy of PH278- and PH62-infected differentiated OTECs. Differentiated OTECs were infected with either pathogenic (PH278) or non-pathogenic (PH62) *M. haemolytica* isolates for the indicated periods of time. Non-adherent bacteria were removed by washing and tissues were fixed in paraformaldehyde. Following permeabilisation, bacteria were immunostained using an anti-*M. haemolytica* whole-cell antibody (green). Host cell cytoskeleton and cilia were stained with anti-β-tubulin (red) and nuclei were stained with DAPI (blue). Samples were mounted and viewed by fluorescence microscopy. (**A**) Wide-field microscopy images of infected OTECs at various time-points post-infection. These demonstrate the formation of invasive foci of PH278 and various stages in the formation of PH62 microcolonies. (**B**) Orthogonal representation of a confocal stack from a PH278-infected culture at 24 h post-infection showing the three-dimensional rounding and sloughing of cells surrounding an invasive focus of infection.

Scanning electron microscopy (Fig. 2) and immunofluorescence microscopy (Fig. 3) proved useful for further characterising the dynamics of tissue colonisation by PH278 and PH62 at a cellular level. While only very infrequent tissue-adherent bacterial cells were observed at 2 h post-infection, large foci of infection were observed from 24 h onwards (Figs. 2A, 3A). The induction of tissue damage and cellular sloughing observed in Fig. 1B was also readily visible by scanning electron microscopy (SEM) (Fig. 2B) and immunofluorescence microscopy (Fig. 3B), primarily in the regions surrounding invasive foci of infection of PH278. Cells of PH278 were predominantly localised beneath the surface of the epithelium, indicating invasive colonisation consistent with our previous reports. The formation of discrete microcolonies of PH62 was clearly visible from 48 h post-infection (Figs. 2B, 3A), whereas no such features were observed for PH278. Microcolonies appeared to be covered in amorphous material reflective of exopolysaccharide production in bacterial biofilms (Fig. 2A). The composition of this material was not confirmed experimentally. Microcolonies of PH62 were remarkably discrete and well-isolated with some inserts harbouring only two/three microcolonies. As such, in spite of consistently high colonisation levels (Fig. 1A), histological sections occasionally appeared negative for PH62 colonisation, even at later stages of infection. The tissue surrounding PH62 microcolonies appeared well-ciliated and healthy unlike that surrounding regions of invasion by PH278.

**Figure 4:**
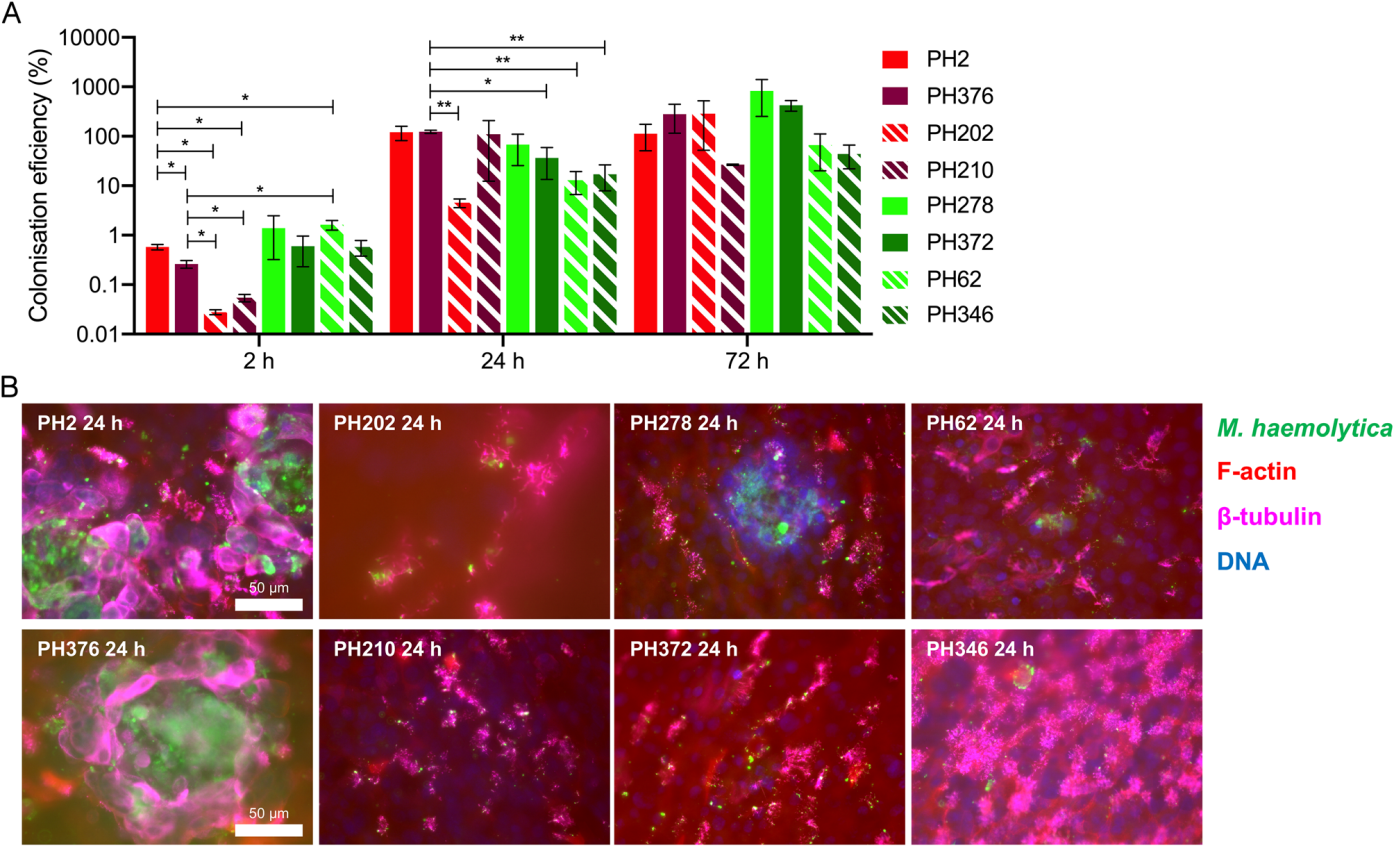
Differentiated OTECs support the colonisation of a wide variety of *M. haemolytica* isolates including pathogenic and non-pathogenic strains. Differentiated OTECs were infected with the indicated *M. haemolytica* isolates for 2 h, 24 h and 72 h. Non-adherent bacteria were removed by washing and tissues were processed as follows. (**A**) Tissues were lysed by addition of 1% (v/v) Triton X-100 and bacteria were enumerated by serial dilution and spot-plating. The numbers of bacteria in the lysate were expressed as a percentage of the inoculum (colonisation efficiency [%]). Columns display means of three experiments +/− standard error, with colonisation efficiency being enumerated from triplicate inserts in each experiment. Bovine isolates are indicated by red columns, while ovine isolates are indicated by green columns. Solid colour indicates pathogenic isolates while dashed columns indicate non-pathogenic isolates. Statistical significance of differences between strains at each independent time-point was assessed by Welch’s unequal variance *t* test with multiple comparisons. Significance values of * and ** represent *p* < 0.05 and 0.01, respectively. (**B**) Tissues were fixed in paraformaldehyde. Following permeabilisation, bacteria were immunostained with an anti-*M. haemolytica* whole-cell antibody (green). Cilia and cytoskeletal microtubules were stained with anti-β-tubulin (magenta), host cell actin was stained with phalloidin Alexa-568 (red) and nuclei were stained with DAPI (blue). Samples were mounted and viewed by wide-field microscopy. In healthy regions of tissue, β-tubulin is concentrated in the apical cell surface-localised cilia while F-actin staining allows visualisation of the underlying epithelium. Upon formation of invasive foci of infection with pathogenic strains, this localisation and normal cellular morphology becomes disrupted. By comparison, with non-pathogenic strains and PH372 only sporadic aggregates of cilia-attached bacteria were observed.

Having observed that differentiated OTECs could support the colonisation of both pathogenic and non-pathogenic ovine isolates of *M. haemolytica*, we next assessed whether isolates associated with disease in cattle could also colonise the tissues. Bovine pathogenic serotype A1 and A6 isolates (PH2 and PH376, respectively) and bovine non-pathogenic serotype A2 isolates (PH202, PH210) were tested for colonisation of OTECs alongside ovine disease-causing serotype A2 isolates (PH278, PH372) and ovine serotype A12 isolates (PH62, PH346) that are rarely associated with disease, in order to robustly assess the strain-specificity of the infection model (Table 1; Fig. 4). These isolates represent part of our laboratory collection, obtained through diagnostic screening over several years, and having been investigated to establish the clonal, serotype and host-specific nature of *M. haemolytica* infections^16,29–31^. In selecting these eight isolates, it was intended that host species and disease status, together with isolate pathogenicity and genetic relatedness/serotype could all be interrogated for a role in OTEC colonisation. All isolates colonised the differentiated OTECs at 2 h, 24 h and 72 h with between 0.028 (PH202, 2 h) and 828.644% (PH278, 72 h) of the inoculum being recovered (Fig. 4A). Colonisation levels were statistically equivalent for all strains at each time-point except for PH2 and PH376 at 2 h, which displayed significantly higher initial adherence/colonisation efficiency than the non-pathogenic bovine isolates PH202 and PH210 and lower initial adhesion than PH62 (PH2 was also found to have significantly higher initial adhesion than PH376 at 2 h), and PH376 at 24 h which displayed significantly higher colonisation efficiency than PH202, PH372, PH62 and PH346 (Fig. 4A). The following trend of decreasing colonisation efficiency at 72 h was observed: ovine pathogenic (PH278 = 828%; PH372 = 425%) > bovine pathogenic (PH2 = 113%; PH376 = 281%) > ovine non-pathogenic (PH62 = 66%; PH346 = 44%). The data obtained for the bovine non-pathogenic isolates (PH202 = 288%; PH210 = 27%) was more variable and as such, the testing of further bovine non-pathogenic isolates would be required in order to discriminate where these isolates fit on this trend. As with the more extensive time-course (Fig. S1), a good deal of overlap was observed between colonisation levels on OTEC cultures derived from independent animals (Fig. S2), highlighting reproducibility in the model.

Immunofluorescence microscopy revealed that bovine pathogenic strains produced invasive foci of infection similar to those induced by PH278 with localised tissue damage and cellular rounding evidenced by cytoskeletal reorganisation and condensation in the OTECs adjacent to these foci (Fig. 4B). In healthy tissues, β-tubulin staining allows for visualisation of the apical surface-localised cilia, while F-actin staining allows for visualisation of the underlying epithelium^24^. This pattern of cytoskeletal organisation and cellular morphology becomes dramatically disrupted in the regions surrounding invasive foci of pathogenic strains (Fig. 4B). By contrast, tissues infected with non-pathogenic isolates of bovine and ovine origin showed disperse regions of bacterial immunostaining, predominantly co-localised with cilia (Fig. 4B). This was surprising given that these isolates colonise efficiently at 24 h, however it should be noted that the invasive foci observed with PH2, PH376 and PH278 were few in number and as such, the greatest contribution to the cell-associated bacterial numbers may be the discrete aggregates associated with the cilia at these time-points. Indeed, PH372 showed no foci of infection in these experiments despite being of very close genetic relatedness to PH278. These results demonstrate that while all *M. haemolytica* isolates are capable of colonising differentiated OTECs at least to some extent, there are differences in the cellular patterns of colonisation and some ovine strains may display enhanced initial adhesion or long-term persistence/proliferation within the model.

## Discussion

During gaseous exchange, the mammalian conducting airways are repeatedly exposed to potentially harmful agents. In the case of intensive farming of livestock, this is particularly problematic as infectious respiratory pathogens can circulate rapidly through populations^32^. One of the primary functions of the respiratory epithelium is to function as an innate immune barrier to invading pathogens. The complex cellular architecture of this tissue plays a role in such defences by physically occluding passage of bacteria and viruses^33^, entrapping particles in goblet cell-secreted mucus^34^ and clearing mucus globules via the coordinated beating of ciliated epithelial cells^35^. Submerged monolayer cell cultures fail to recapitulate these fundamental processes^25,36,37^, and therefore are poorly suited to the study of colonisation by pathogenic species. We recently developed well-differentiated primary OTECs for use in modelling interactions of relevant pathogens with the sheep respiratory epithelium in vitro^24,25^. The model forms a heavily ciliated, mucus-producing cell layer with similar physiology to that of the native respiratory tissue. In tandem, we have developed a BBEC culture model which well represents the respiratory epithelium of cattle^26,27^.

The sero-specific nature of severe cases of respiratory disease by *M. haemolytica*^11,38^, coupled with a suggested role for the polysaccharide capsule (on which the serotyping classification is based) in cell adhesion^17^, indicates that isolates may be specifically adapted to colonising the airway tissues of discrete hosts. For example, sheep displaying severe respiratory disease are often colonised heavily by *M. haemolytica* serotype A2 but rarely by serotype A12^13,16^. However, capsular polysaccharide type by itself is not likely to be the only factor associated with host-specificity and virulence. Indeed, we have previously demonstrated specific associations of *lktA*^39^, *ompA*^29^ and *tbpA* and *tbpB* alleles^31^ with *M. haemolytica* lineages or clones associated with either cattle or sheep. We previously put forward evidence to support the hypothesis that OmpA, a major surface-exposed outer membrane protein, is under strong selective pressure from the host species and plays an important role in host-adaptation^29^. It was subsequently shown that OmpA plays a role in adherence of *M. haemolytica* to bovine airway epithelial cells^19^. Thus, in vitro differentiated cattle and sheep airway epithelial cell culture models represent an excellent opportunity to study the molecular basis of such host-specificity. On comparison of *M. haemolytica* PH2 (a pathogenic bovine serotype A1 isolate) with PH202 (a non-pathogenic serotype A2 isolate) colonisation on differentiated BBECs, it was observed that only PH2 was capable of proliferation beyond 12 h post-infection, whereas PH202 was completely cleared from the majority of BBEC cultures^28^. We hypothesised that this may have been due to susceptibility of PH202 to BBEC-produced antimicrobial compounds such as defensins and cathelicidins, which are well-defined innate defence mechanisms of the respiratory epithelial cells^40,41^. Pathogenic isolate PH2 invaded through the apical surface of the BBEC layer, proliferated intracellularly and paracellularly, resulting in a drop in trans-epithelial electrical resistance and a break down in the epithelial barrier. By contrast, in this study both PH278 (a pathogenic ovine serotype A2 isolate) and PH62 (a non-pathogenic ovine serotype A12 isolate) were capable of colonising differentiated OTECs throughout the duration of the time-course, albeit with higher levels being observed for PH278 at time-points post 16 h (Fig. 1). Viable counts of PH62 were recovered from infected OTECs at all time-points (Fig. 1) indicating that antimicrobial-induced killing of this commensal isolate did not occur.

Pathogenic isolate PH278 caused the formation of similar foci of infection in OTECs to those observed with PH2 in BBECs, with invasive proliferation and tissue disruption being observed (Figs. 1, 2, 3). PH62 produced a previously unseen phenotype during colonisation, with the formation of large microcolonies within the infected tissue (Figs. 2B, 3A). The superficial differences in the pattern of adherence between pathogenic and non-pathogenic isolates observed here may reflect important attributes of the behaviour of these strains in vivo. For example, pathogenic isolates such as PH278 may engage in unchecked replication, leading to extensive tissue disruption, while non-pathogenic isolates form discrete microcolonies with minimal disruption to the surrounding epithelium.

In general, across the eight strains tested, a trend towards increasing colonisation by pathogens, particularly ovine pathogens, was observed (Fig. 4A). This trend is likely reflective of the enhanced proliferative capacity of pathogenic *M. haemolytica* isolates. However, there were marked differences in the colonisation efficiencies of the bovine serotype A2 isolates PH202 (288%) and PH210 (27%) at 72 h. Although these isolates are representative of the same electrophoretic type (ET) and would be expected to perform similarly, there may be subtle differences between them (they do have slightly different OMP-profiles—see Table 1) which could account for these observations. Klima et al.^10^ suggested the existence of a distinct subset of serotype A2 strains that are adapted for proliferation in immuno-compromised bovine hosts and the observed differences in colonisation between these two isolates might reflect such strain variation. The analysis of further strains would help resolve this but, importantly and as we have demonstrated, the differentiated airway epithelial cell model described herein has the capability of discriminating between such strains without the need for in vivo challenge experiments. It should be noted that sloughing of tissue from OTECs heavily infected with pathogenic *M. haemolytica* at 72 h could result in loss of enumerable bacterial cells, thereby reducing the difference in colonisation efficiency between pathogenic and non-pathogenic isolates and blurring the significance of differences between these groups.

The stark contrast between the colonisation efficiency of the non-pathogenic isolates of bovine (PH202) and ovine (PH62) origin on tissues from their respective animals of isolation is interesting. It may reflect variability in the colonisation factor repertoire of the isolates themselves, or receptor variation in the differentiated BBECs and OTECs. We chose to use bronchial tissue of bovine origin due to enhanced differentiation over bovine tracheal tissue in our hands and tracheal tissue of ovine origin due to difficulty encountered in obtaining sufficient bronchial cell numbers for large scale experimentation (without the need for extensive passage, which might compromise differentiation capacity). Therefore, it is possible that non-pathogenic isolates are specifically capable of colonising upper airway epithelial cells in vitro. A four-way comparison of upper (tracheal/nasal) and lower (bronchial/bronchiolar) airway tissues would prove useful in dissecting these potential tissue tropisms. The possession of models which allow for the study of commensal interactions as well as pathogenic interactions with *M. haemolytica* will prove useful in uncovering the mechanisms by which *M. haemolytica* switches between these two important lifestyles.

Cattle and sheep display susceptibility to discrete clonal groups of *M. haemolytica*^16^. We assessed whether interactions with differentiated OTECs could reflect this species-specificity by analysing colonisation of pathogenic and non-pathogenic isolates of both bovine and ovine origin. All of the isolates tested were able to colonise and proliferate within the differentiated OTEC layer (Fig. 4). It is therefore apparent that the host-specificity of *M. haemolytica* does not arise at the level of colonisation (at least within differentiated OTECs). As such, there appears to be an inherent lack of specificity associated with colonisation of OTECs in direct contrast to the selectivity we have observed for BBECs^28^, highlighting the potential for variation in the outcome of infection experiments performed using tissue culture models of different origins. It has been established that leukotoxin is the primary virulence factor of *M. haemolytica* contributing greatly to the fibrinous necrotising pneumonia observed in infected animals. As leukotoxin binding is highly species-specific^42^ and cattle and sheep isolates tend to encode different *lktA* alleles^39^, it is possible that host-specificity occurs downstream of colonisation and is mediated by virulence factors that are not involved in attachment. However, the possibility of more species and pathotype specific interactions occurring exclusively with tissues from deeper in the respiratory tract should not be excluded.

Finally, the experiments conducted herein involve pure culture infections with perhaps the most important pathogen of the bovine respiratory disease complex, *M. haemolytica*. However, we are currently exploring how further levels of complexity in the modelling of this disease may advance our understanding. For example, three individual viral components of the respiratory disease complex have been shown to replicate efficiently within differentiated BBECs^43^. We are interested in how such infections might affect the outcome of a subsequent infection with *M. haemolytica*. As mentioned previously, the progression of respiratory disease in ruminants is not exclusively dependent on epithelial interactions but also interactions with immune cells. The addition of neutrophils or macrophages to our infection model would allow for analysis of resistance to phagocytosis and importantly cytolysis and degranulation events stimulated by leukotoxin. Overall, we are optimistic that continued study with these models will lead to a significant enhancement of our understanding of ruminant respiratory disease at a molecular level.

## Methods

### Bacterial strains and culture conditions

A panel of pathogenic and non-pathogenic *M. haemolytica* isolates of bovine and ovine origin and known capsular serotypes were chosen for inclusion in this study. The isolates were selected to represent specific ETs which reflected their genetic relatedness^16^. Bovine pathogenic isolates were represented by serotype A1 (PH2) and A6 (PH376) isolates of ET1 that had been recovered from the lungs of pneumonic cattle; bovine non-pathogenic isolates were represented by serotype A2 (PH202 and PH210) isolates of ET21 that had been recovered from the nasopharynxes of healthy animals. Ovine pathogenic isolates were represented by serotype A2 (PH278 and PH372) isolates of ET21 that had been recovered from the lungs of pneumonic sheep; ovine non-pathogenic isolates were represented by serotype A12 (PH62 and PH346) isolates of ET1, of which PH62 had been recovered from the nasopharynx of a healthy lamb. Most of these strains have been characterised with respect to their outer membrane protein (OMP)- and lipopolysaccharide (LPS)-types^44^ and their *lktA*, *lktCABD*, *ompA* and *tbpBA* allele types^29–31,39^. Properties of the strains are shown in Table 1. Bacteria were routinely cultured on brain heart infusion (BHI) agar containing 5% (v/v) defibrinated sheep’s blood. Overnight cultures were prepared by inoculating several colonies in BHI and culturing at 37 °C, 120 rpm for approximately 16 h. All reagents were purchased from Sigma-Aldrich unless otherwise specified.

### Generation of differentiated OTEC cell cultures

Cultures of differentiated OTECs were prepared as previously described^24,25^. Briefly, OTECs were extracted from tracheal epithelia using protease XIV from *Streptomyces griseus*, at 4 °C overnight. Digestion was halted by the addition of foetal calf serum (FCS) and loosely bound epithelial cells were removed by vigorous washing. The cells were strained through a 70 μm strainer, centrifuged at 300 × *g* for 5 min and washed with serum-containing growth medium (SGM), which comprised a 1:1 mixture of DMEM/Ham’s F12 with 10% (v/v) FCS, 1% (v/v) Penicillin–Streptomycin and 1% (v/v) Fungizone. Cell pellets were resuspended in airway epithelial growth medium (AEGM [Promocell, #C-21160]) containing 1% (v/v) Pen-Strep and 1% (v/v) Fungizone (Thermofisher Scientific) and seeded into T75 cell culture flasks. Flasks were routinely incubated at 37 °C in 5% CO_2_ and 14% O_2_. When flask cultures reached ~ 70% confluency (4–6 days), OTECs were removed by trypsinisation, washed and seeded onto Greiner 0.4 μm pore-diameter Thincerts (#665640) pre-coated with bovine type I collagen (Becton Dickinson) according to the manufacturer’s instructions. A seeding density of 2.5 × 10^5^ cells per insert was employed. Submerged culture in AEGM at 37 °C, 5% CO2 and 14% O_2_ was allowed to proceed until confluency was reached and a trans-epithelial electrical resistance (TEER) of at least 200 Ω × cm^2^ was achieved (5–10 days). The OTECs were gradually transitioned to ALI medium (DMEM/AEGM base medium [1:1] supplemented with the following growth factors: 10 ng ml^−1^ epidermal growth factor [EGF], 100 nM retinoic acid [RA], 6.7 ng ml^−1^ triiodothyronine [T3], 5 μg ml^−1^ insulin, 500 ng ml^−1^ hydrocortisone, 500 ng ml^−1^ epinephrine and 10 μg ml^−1^ transferrin) by feeding apically and basally with a 1:1 mix of AEGM/ALI medium approximately half-way through the submerged growth phase. After 200 Ω × cm^2^ TEER was observed, the cells were washed with phosphate buffered saline (PBS) and fed from the basal compartment only with ALI medium. Feeding and washing were carried out every 2–3 days throughout submerged and ALI growth. A total of 21 days growth at ALI was allowed before OTECs were considered fully differentiated and ready for infection.

### Infection of differentiated OTECs

Twenty-four hours prior to infection, the OTECs were washed apically and basally before feeding with antibiotic-free ALI medium basally (to allow the cells to equilibrate and produce mucin prior to infection). Bacteria were cultured to exponential phase (approximately 4 h 30 min) before harvesting by centrifugation, washing and resuspending in PBS. The OD_600 nm_ was recorded and the cell suspension was adjusted to 1.0 × 10^9^ cells ml^−1^. Twenty-five microlitres of this suspension (2.5 × 10^7^ CFU) were used to infect each differentiated cell culture insert. Infection was carried out at 37 °C in 5% CO_2_ and 14% O_2_ for the indicated periods of time. At each time-point, tissues were washed three times with PBS and tissues were analysed by lysis and plate counting, histological analysis, immunofluorescence microscopy and SEM.

### Assessment of colonisation efficiency

Inocula used for infection of differentiated OTECs were titered by serial dilution and spot-plating. Infected tissues were lysed by incubating in 1% (v/v) Triton X-100 in PBS for 15 min before scraping from the cell culture insert and homogenising by repeated pipetting. Lysates were titered as with inocula. Colonisation efficiency was calculated by expressing the numbers of CFU in the lysate at a given time as a percentage of the inoculum. Three independent inserts were used for each experiment and three experiments with tissue from independent animals were carried out.

### Histological and immunohistochemical processing and analysis

OTEC cultures were fixed with 4% (w/v) paraformaldehyde for 15 min. Samples were washed with PBS and dehydrated through increasing ethanol concentrations. The tissues were cleared with xylene, infiltrated with paraffin wax and embedded in wax blocks. Sections (2.5 μm thickness) were cut using a Thermoshandon Finesse ME + microtome and stained with H&E using standard histological procedures. For immunohistochemical localisation of bacteria, tissues were processed with a Menarini Antigen Access Unit. Endogenous peroxide was blocked using H_2_O_2_ before incubating in rabbit anti-OmpA^PH278^(^45^) at a 1 in 800 dilution for 30 min. Primary antibodies were labelled using an anti-mouse-HRP polymer and detected using REAL EnVision Peroxidase/DAB + Detection System (Dako, #K3468) according to the manufacturer’s instructions. Sections were counter-stained using Gill’s haematoxylin, dehydrated, cleared and mounted in synthetic resin. Samples were visualised using a Leica DM2000 microscope.

### Immunofluorescence microscopy

Immunofluorescence microscopy was carried out as described previously^24,25^ with some alterations. Infected OTEC cultures were fixed by incubating in 4% (w/v) paraformaldehyde for 15 min. The samples were washed with PBS and permeabilised in permeabilisation buffer (PBS containing 0.5% [v/v] Triton X-100, 100 mg ml^−1^ sucrose, 4.8 mg ml^−1^ HEPES, 2.9 mg ml^−1^ NaCl and 600 μg ml^−1^ MgCl_2_, pH 7.2) for 10 min. Following three washes with PBST (PBS containing 0.05% [v/v] Tween-20), tissues were blocked in blocking buffer (PBST containing 10% normal goat serum and 1% [w/v] BSA) for 1 h. The samples were washed three times with PBST and incubated in primary antibody diluted in blocking buffer for 1 h. Rabbit anti-β-tubulin (Abcam, #ab6046) was used at a 1:200 dilution, while bovine anti-*M. haemolytica* whole-cell antibody was used at a 1:50 dilution. The samples were washed three times and incubated in secondary antibody diluted in blocking buffer for 1 h. Goat anti-rabbit Alexa Fluor 488 (Thermofisher, #A-11034) and goat anti-bovine FITC (Thermofisher, #A18752), were used at a 1:400 dilution. The samples were washed three times and stained with rhodamine phalloidin (1U per sample) and 30 femtomoles DAPI for 20 min. Following three final washes with PBS, the membranes were cut from the inserts and mounted in Vectashield. Wide-field images were acquired using a Leica Dmi8 microscope. Confocal images were acquired using a Zeiss LSM510.

## Scanning electron microscopy

Infected OTEC cultures were fixed with 1.5% (v/v) glutaraldehyde in 0.1 M sodium cacodylate at 4 °C for 1 h. All subsequent incubations were carried out at room temperature. The samples were then washed three times with 0.1 M sodium cacodylate and incubated in 2% (w/v) osmium tetraoxide for 1 h. Following three washes with distilled water, the tissues were incubated for 1 h in 0.5% (w/v) uranyl acetate while protected from light. After two additional washes, the tissues were dehydrated via a series of increasing ethanol concentrations and incubated in hexamethyldisilizane for 1 h before drying overnight in a desiccator. The tissue samples were mounted on aluminium SEM stubs using carbon tape and sputter-coated with gold. Images were acquired using a Jeol 6400 electron microscope. Bacteria were manually false-coloured yellow using Photoshop Elements.

### Statistical analysis

Data were analysed using GraphPad Prism 8. Each experiment was performed using tissues isolated from independent animals, with three inserts being employed for quantitation of colonisation efficiency at each time-point. Triplicate experimental means were used for statistical analysis. For each independent time-point, strains were compared by two-tailed Student’s *t* test or Welch’s unequal variance *t* test with multiple comparisons as indicated. Significance values of *, ** and *** represent *p* < 0.05, 0.01 and 0.001, respectively.

## Supplementary information

Supplementary information.
