# Supplementary material for: Differentiated ovine tracheal epithelial cells support the colonisation of pathogenic and non-pathogenic strains of Mannheimia haemolytica
Source: Sci Rep. 2020 Sep 11;10:14971. doi: 10.1038/s41598-020-71604-8 (PMC7486916; doi:10.1038/s41598-020-71604-8)
Supplement: Supplementary file 1 — Supplementary information. [file 41598_2020_71604_MOESM1_ESM.docx]

**Supplementary Information**

**Differentiated ovine tracheal epithelial cells support the colonisation of pathogenic and non-pathogenic strains of *Mannheimia haemolytica***

Nicky O’Boyle^1^, Catherine C Berry^2^ and Robert L Davies^1^*

^1^Institute of Infection, Immunity and Inflammation, College of Medical, Veterinary and Life Sciences, University of Glasgow, Glasgow, United Kingdom

^2^Institute of Molecular, Cell and Systems Biology, College of Medical, Veterinary and Life Sciences, University of Glasgow, Glasgow, United Kingdom

*Corresponding Author: robert.davies@glasgow.ac.uk


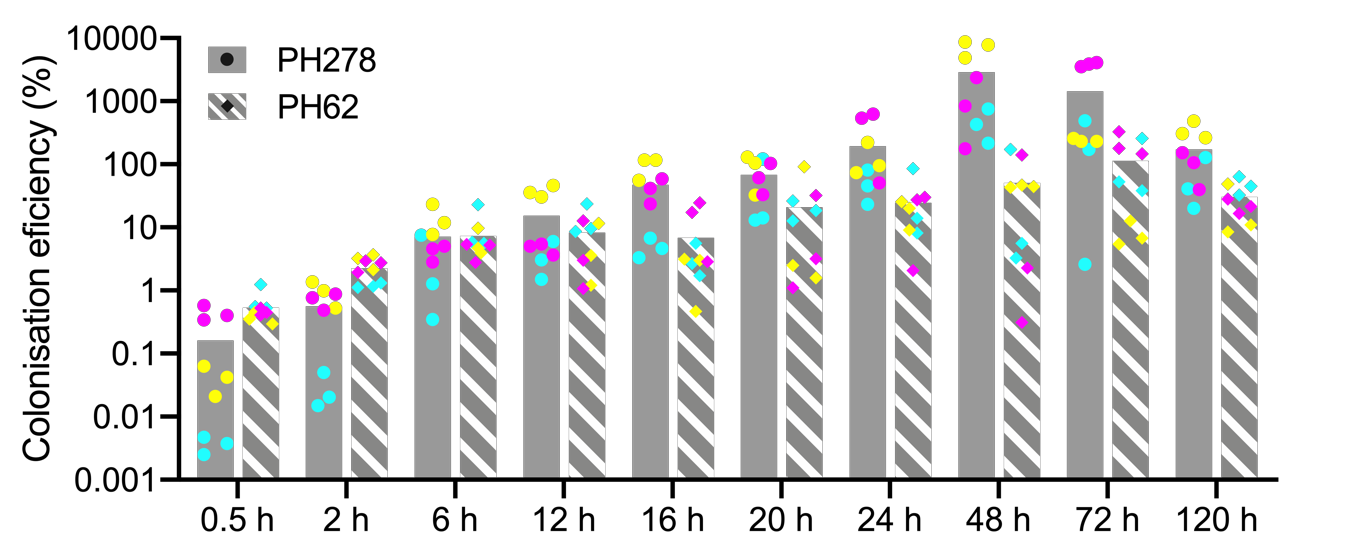


**Fig. S1.** **Reproducible levels of colonisation by PH278 and PH62 were observed on tissues derived from independent animals.** Differentiated OTECs were infected with either pathogenic (PH278) or non-pathogenic (PH62) *M. haemolytica* isolates for the indicated periods of time. Non-adherent bacteria were removed by washing, tissues were lysed by addition of 1% (v/v) Triton X-100 and bacteria were enumerated by serial dilution and spot plating. The numbers of bacteria in the lysate were expressed as a percentage of the inoculum (colonisation efficiency [%]). Columns display means of three experiments. Three independent cell culture inserts were used per experiment. The colonisation efficiency observed on each insert is displayed by the coloured data points, with colouring (cyan – animal 1, yellow – animal 2, magenta – animal 3) representing tissues derived from independent animals.


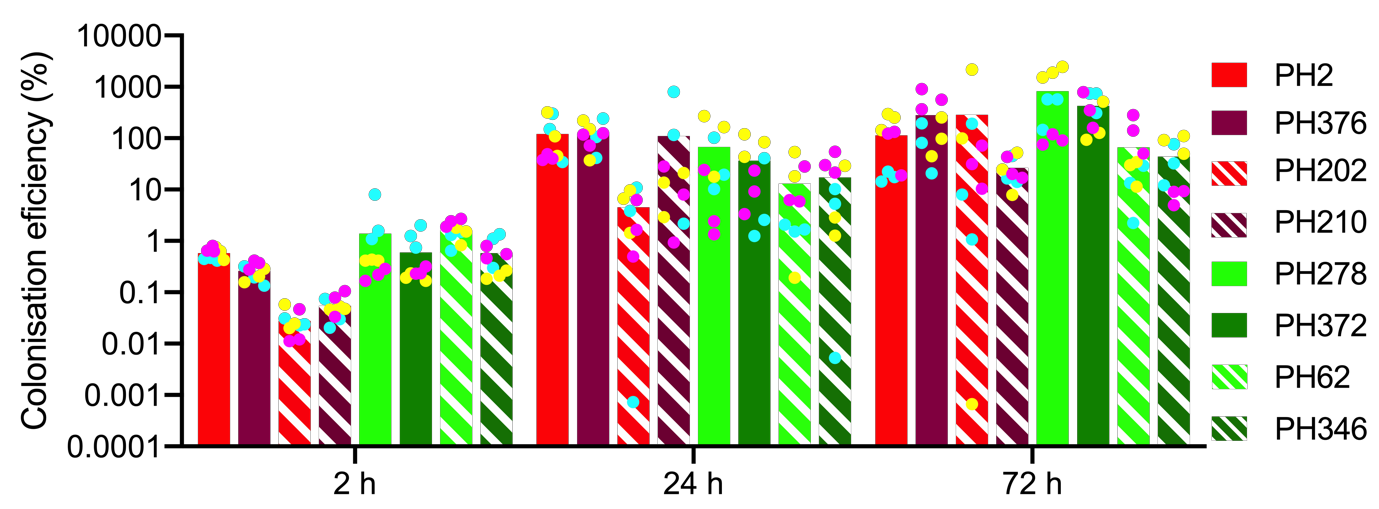


**Fig. S2.** **Reproducible levels of colonisation by eight strains were observed on tissues derived from independent animals.** Differentiated OTECs were infected with each of eight *M. haemolytica* isolates for the indicated periods of time. Non-adherent bacteria were removed by washing, tissues were lysed by addition of 1% (v/v) Triton X-100 and bacteria were enumerated by serial dilution and spot plating. The numbers of bacteria in the lysate were expressed as a percentage of the inoculum (colonisation efficiency [%]). Columns display means of three experiments. Three independent cell culture inserts were used per experiment. The colonisation efficiency observed on each insert is displayed by the coloured data points, with colouring (cyan – animal 1, yellow – animal 2, magenta – animal 3) representing tissues derived from independent animals.
